# Neural stem cells derived from the developing forebrain of YAC128 mice exhibit pathological features of Huntington’s disease

**DOI:** 10.1111/cpr.12893

**Published:** 2020-08-31

**Authors:** Endan Li, Hee Ra Park, Chang Pyo Hong, Younghoon Kim, Jiwoo Choi, Suji Lee, Hyun Jung Park, Bomi Lee, Tae Aug Kim, Seong Jin Kim, Hyun Sook Kim, Jihwan Song

**Affiliations:** ^1^ Department of Biomedical Science CHA Stem Cell Institute CHA University Seongnam‐si Korea; ^2^ iPS Bio, Inc. Seongnam‐si Korea; ^3^ Theragen Etex Bio Institute Suwon‐si Korea; ^4^ Department of Neurology CHA Bundang Medical Center CHA University Seongnam‐si Korea

**Keywords:** drug screening, Huntington's disease, mutant Huntingtin, neural stem cells, YAC128 transgenic mice

## Abstract

**Objectives:**

Huntington's disease (HD) is a devastating neurodegenerative disease caused by polyglutamine (polyQ) expansion in the huntingtin (HTT) gene. Mutant huntingtin (mHTT) is the main cause of HD and is associated with impaired mitochondrial dynamics, ubiquitin‐proteasome system and autophagy, as well as tauopathy. In this study, we aimed to establish a new neural stem cell line for HD studies.

**Materials and methods:**

YAC128 mice are a yeast artificial chromosome (YAC)‐based transgenic mouse model of HD. These mice express a full‐length human mutant HTT gene with 128 CAG repeats and exhibit various pathophysiological features of HD. In this study, we isolated a new neural stem cell line from the forebrains of YAC128 mouse embryos (E12.5) and analysed its characteristics using cellular and biochemical methods.

**Results:**

Compared to wild‐type (WT) NSCs, the YAC128 NSC line exhibited greater proliferation and migration capacity. In addition to mHTT expression, increased intracellular Ca^2+^ levels and dysfunctional mitochondrial membrane potential were observed in the YAC128 NSCs. YAC128 NSCs had defects in mitochondrial dynamics, including a deficit in mitochondrial axonal transport and unbalanced fusion and fission processes. YAC128 NSCs also displayed decreased voltage response variability and Na^+^ current amplitude. Additionally, the ubiquitin‐proteasome and autophagy systems were impaired in the YAC128 NSCs.

**Conclusions:**

We have established a new neural stem line from YAC128 transgenic mice, which may serve as a useful resource for studying HD pathogenesis and drug screening.

## Graphical abstract

The YAC128 NSC, a new neural stem cell line derived from the developing forebrain of YAC128 mice, exhibits pathological features of Huntington's disease, including mHTT aggregation, defective ubiquitin and autophagy systems and impaired axonal transport of mitochondria, which can serve as a useful resource for studying HD pathogenesis and drug screening

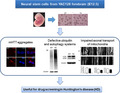

## 1 INTRODUCTION

Huntington's disease (HD) is an autosomal dominant neurodegenerative disorder caused by an expanded polyglutamine (polyQ) tract in exon 1 of the huntingtin gene on the short arm of chromosome 4. It is characterized by chorea, psychiatric disturbance and cognitive decline.^1^, ^2^, ^3^ HD is associated with the formation of aggregates containing mutant huntingtin (mHTT) gene products, which are the main cause of HD progression. In addition, transcriptional dysregulation, mitochondrial abnormalities, defective cytoskeleton and axonal transport, reduced neurotrophic factor production, oxidative stress, ubiquitin‐proteasomal dysfunctions and altered autophagy^4^, ^5^, ^6^ have also been implicated in HD progression. Several mouse neural stem cell models of HD have been characterized previously,^7^, ^8^, ^9^, ^10^, ^11^, ^12^ but more reliable cellular models for drug screening are needed.

The YAC128 transgenic (TG) mouse is a yeast artificial chromosome (YAC)‐based transgenic mouse model which expresses the full‐length human mutant HTT gene with 128 CAG repeats^13^ and shows a progressive decline in motor function.^13^, ^14^ Since the YAC128 mouse represents various aspects of HD pathology relatively well, it has been one of the most commonly used HD mouse models thus far. Recently, it has been reported that adult neural progenitor cells isolated from the subventricular zone of the YAC128 mouse brain exhibit typical HD phenotypes, including enhanced calcium and ROS signals and increased proliferation and migration.^15^ Embryonic fibroblasts derived from YAC128 mice also display increased calcium signalling and superoxide generation.^16^

In this study, we established a neural stem cell line from the developing forebrain of YAC128 TG mice (E12.5) and characterized its pathological phenotypes. Our results strongly suggest that this new neural stem cell line is a potentially useful tool for the study of HD pathogenesis and drug screening.

## 2 MATERIALS AND METHODS

### 2.1 Derivation of neural stem cells from the developing forebrain of E12.5 YAC128 mice

YAC128 HD transgenic mice were purchased from the Jackson Laboratory. The generation and breeding of transgenic YAC128 HD mice (FVBN/NJ background strain) have been described previously.^13^ Neural stem cells^17^ were isolated from the forebrain of E12.5 YAC128 TG and wild‐type embryos. Isolation of NSCs was performed as described previously, with minor modifications.^15^, ^18^ Briefly, the forebrain regions were isolated and minced into small pieces for 1‐2 minutes and then treated with 0.5% trypsin‐EDTA for 5 minutes at 37°C. Cells were dissociated in DMEM basal medium containing 10% FBS and 0.1 mg/mL penicillin/streptomycin and then plated on tissue culture dishes coated with poly‐L‐ornithine (PLO; 15 µg/mL, Sigma‐Aldrich) and fibronectin (FN; 1 µg/mL, Sigma‐Aldrich). The next day, the culture medium was replaced with NSC complete medium, which consisted of DMEM/F12 containing 100 × N2 supplement, 300 mmol/L D‐glucose, 200 mmol/L L‐glutamine, 100 U/mL penicillin, 100 µg/mL streptomycin, 0.1 mmol/L non‐essential amino acids, 0.1 mmol/L β‐mercaptoethanol, 10 ng/mL epidermal growth factor (EGF) and 10 ng/mL basic fibroblast growth factor (bFGF). Once cell growth was stable, we performed magnetic sorting using PSA‐NCAM^+^ microbeads (Miltenyi Biotec), which allowed us to obtain a pure population of neural stem cells. TrypLE Select (Thermo Fisher Scientific, MA) was used for splitting the cells. We maintained the neural stem cells at 37°C in a humidified incubator with 5% CO_2_ and passaged the cells every 3 days.

### 2.2 Neuronal differentiation of YAC128 NSC

To differentiate the neural stem cells into mature neurons, we plated cells directly onto PLO/Laminin (5 µg/mL, Sigma‐Aldrich)‐coated dishes and four well glasses. We used a modified version of an existing protocol,^11^ which requires the withdrawal of EGF and slow reduction of FGF‐2. Briefly, the differences are as follows: 1:1 DMEM/F12 neurobasal medium was used as the basic differentiation medium in place of EUROMED‐N medium. The differentiation medium consisted of 200× N2 supplement, 100× B27, 100 U/mL penicillin and streptomycin, 0.1 mmol/L non‐essential amino acids, 0.1 mmol/L β‐mercaptoethanol, 3 mmol/L D‐glucose and 2 mmol/L L‐glutamine. Additionally, NGF was used for neuronal induction in place of BDNF. Based on these modifications, we supplemented the differentiation medium with 20 ng/mL NGF and 10 ng/mL FGF‐2 for the first 3 days, and then we added 30 ng/mL NGF and 6.7 ng/mL FGF‐2 for the next 3 days. Afterwards, the cells were maintained in differentiation medium supplemented with 30 ng/mL NGF and 5 ng/mL FGF‐2 for an additional 21 days.

### 2.3 Genomic DNA isolation and PCR genotyping

Genomic DNA was isolated from the tail and NSCs of E12.5 embryos using genomic DNA isolation buffer (10 mmol/L Tris‐HCl, pH 8.0, 200 mmol/L NaCl, 10 mmol/L EDTA, 0.5% SDS, and 100 μg/mL Proteinase K [Roche]). Briefly, tails and NSCs were incubated in genomic DNA isolation buffer for 5 hours at 55°C. Next, phenol‐chloroform extraction and ethanol precipitation were performed. The purified genomic DNA was dissolved in TE buffer (10 mmol/L Tris‐HCl, pH 8.0, and 1 mmol/L EDTA), and the mutant Htt gene was amplified. PCR amplification was performed using PrimeSTAR premix (Takara Bio). The following primer sequences were used: forward primer 5′‐CCG CTC AGG TTC TGC TTT TA‐3′ and reverse primer 5′‐TGG AAG GAC TTG AGG GAC TC‐3′ (Figure S1A).

### 2.4 Karyotype analysis

Standard G‐banding karyotyping was performed by Korea Research of Animal Chromosomes (Seoul, Korea).

### 2.5 Western blot analysis

Cells were lysed with RIPA buffer (150 mmol/L NaCl, 1% Nonidet P‐40, 0.5% deoxycholic acid, 0.1% SDS, and 50 mmol/L Tris‐HCl, pH 7.4) containing protease inhibitor (Roche) and phosphatase inhibitor (Roche), followed by sonication and incubation on ice for 10 minutes. Next, the cells were centrifuged at 13 200 *g* for 10 minutes. The supernatant was collected, and the protein concentrations were determined by BCA assay (Thermo Fisher Scientific). Equal amounts of proteins were loaded onto 8%~12% SDS polyacrylamide gels. The separated samples were transferred to PVDF membranes and incubated with primary antibodies against the target proteins, followed by incubation with horseradish peroxidase (HRP)‐conjugated goat anti‐mouse IgG or HRP‐conjugated goat anti‐rabbit IgG. Protein bands were visualized by enhanced chemiluminescence (ECL) using a bioimaging analyser (Bio‐Rad Laboratories). The relative intensity of each band was measured using ImageJ software (rsb.info.nih.gov, by W. Rasband). The following primary antibodies were used: anti‐1C_2_ (1:500, Millipore); anti‐mutant huntingtin (EM48; 1:500, Millipore), anti‐Ub (1:5000, Santa Cruz Biotechnology), anti‐LC3B (1:1000, Cell Signaling), anti‐p‐Drp1 (Ser637) (1:1000, Cell Signaling), anti‐Drp1 (1:1000, Cell Signaling), anti‐Fis1 (1:1000, Santa Cruz Biotechnology), anti‐OPA1 (1:1000, Cell Signaling), anti‐Mfn1 (1:1000, Abcam) and anti‐Mfn2 (1:1000, Cell Signaling). β‐actin (1:5000, Santa Cruz Biotechnology) was used as an internal control.

### 2.6 Immunocytochemical analysis

YAC128 NSCs were transferred to 12 mm round cover slips coated with PLO/Laminin in a four‐well plate (BD Biosciences). The cells were then washed with chilled PBS and fixed with 4% paraformaldehyde for 20 minutes at room temperature. Next, the cells were permeabilized and blocked with 0.1% Triton X‐100 (Sigma‐Aldrich) and 5% normal horse serum (Vector Laboratories) diluted in PBS for 30 minutes at room temperature. The cells were then incubated with primary antibody overnight at 4°C, followed by incubation with secondary antibody. The following secondary antibodies were used: Alexa‐555‐conjugated goat anti‐mouse IgG; Alexa‐555‐conjugated goat anti‐rabbit IgG; Alexa‐488‐conjugated goat anti‐rat IgG; and Alexa‐488‐conjugated goat anti‐rabbit IgG (1:200, Molecular Probes). Images were obtained using two confocal laser‐scanning microscope systems: a Zeiss LSM 880 scanning confocal microscope (Zeiss) and a Leica TCS Sp5 II confocal microscope (Leica). The following primary antibodies were used: anti‐NESTIN (1:40, Developmental Studies Hybridoma Bank), anti‐SOX2 (1:200, Millipore) for the detection of uncommitted neural stem cells, anti‐MAP2 (1:500, Millipore), anti‐Tuj1 (1:500, Millipore) for the detection of differentiated neurons, anti‐HTT (1:100, Sigma‐Aldrich) and anti‐ubiquitin (1:50, Santa Cruz Biotechnology).

### 2.7 Mitochondrial membrane potential (MMP) assay

Depolarization of mitochondrial membrane potential was measured using a cationic carbocyanine dye, JC‐1 (Molecular Probes). JC‐1 aggregates in the mitochondria (red) indicate high mitochondrial membrane potential (MMP), and free JC‐1 monomers (green) indicate low MMP in the mitochondria (*ie* mitochondrial depolarization). Images were obtained using a Zeiss LSM 880 confocal microscope and analysed using ImageJ.

### 2.8 Intracellular Ca^2+^ measurement

Cells were treated with a final concentration of 1 µmol/L Fluo‐4 AM (Molecular Probes) for 10 minutes. Subsequently, the cells were washed with culture medium, and intracellular Ca^2+^ (green fluorescence, excitation at 485 nm and emission at 520 nm) was measured every 5 seconds using a Zeiss LSM 880 confocal microscope. The green fluorescence signal was quantified using ImageJ.

### 2.9 Mitochondrial live cell imaging

Differentiated NSCs (DIV7) were incubated with MitoTracker Red (Thermo Fisher Scientific; M7512) at a final concentration of 10 nmol/L for 15 minutes before live cell imaging analysis. Live cell imaging was performed using a Zeiss LSM 880 Meta confocal microscope with an enclosed climate chamber. Cells were maintained at 37°C in 5% CO_2_ /95% air during imaging. To generate kymographs, time‐lapse images were recorded every second for 2 minutes. Quantitative analysis of mitochondria velocity was performed using IMARIS software (BITPLANE). Axons were designated as regions of interest (ROI) in IMARIS. Particles were identified as mitochondria, and all of the mitochondria in a given ROI were tracked. To select the velocity ranges which correctly represented the mitochondrial movement in differentiated NSCs, instantaneous velocities of each mitochondrion at each time point were plotted as a histogram with a 0.5 mm/s interval. ImageJ was used to produce Figure 3A.

### 2.10 Electrophysiological recording

Whole‐cell patch recordings were performed at DIV14, 21 and 28 as previously described.^19^, ^20^ Briefly, before the recordings, the cells were transferred to a Nikon FN2 upright microscope (Optical Apparatus) fitted with a 40× water‐immersion objective, differential interference contrast (DIC) and infrared filter (IR) and perfused in aerated (95% O_2_/5% CO_2_) artificial CSF (ACSF) at room temperature (21‐25°C). The ACSF consisted of 124 mmol/L NaCl, 3 mmol/L KCl, 26 mmol/L NaHCO_3_, 1.3 mmol/L MgSO_4_, 1.25 mmol/L NaH_2_PO_4_, 2.4 mmol/L CaCl_2_ and 10 mmol/L glucose. Micropipettes (tip diameter, 1.5‐2.0 μm; 3‐6 MΩ) were pulled from borosilicate tubing (P‐97; Sutter Instrument) and filled with an intracellular solution containing 143 mmol/L K‐glutamate, 10 mmol/L HEPES, 2 mmol/L KCL and 0.5 mmol/L EGTA, pH 7.2‐7.3 with KOH. Recordings were performed using an EPC‐10 USB patch‐clamp amplifier (EPC‐10 USB, HEKA Elektronik). Voltage control, current recording and filtration of current (at 1 kHz) were obtained using an EPC‐10 patch‐clamp amplifier (EPC‐10, HEKA Elektronik) linked to a PC with HEKA software. After the rupture of the cell membrane, leak conductance subtraction and series resistance compensation (70%‐80%) were performed and monitored periodically. In the voltage‐clamp (VC) configuration, cells were subjected to a series of voltages (duration, 50 ms) from −90 to +30 mV from a holding potential of −60 mV. In the current‐clamp (CC) configuration, resting membrane potential was measured, and then a negative holding direct current (in the range: −2 to −15 pA) was applied to bring the membrane potential to approximately −60 mV. This was done to compare neurons under identical conditions during AP firing. To assess the AP firing pattern in the current‐clamp configuration, we applied a series of currents (duration, 800ms) from −20 to +60 pA. Current intensities were modified depending upon the input resistance of cells. Peak sodium current is defined as maximal transient inward current at any command voltage. Peak potassium current was measured at 40 ms from the onset of the command voltage pulse. Summary data are shown as mean ± standard error of the mean. Statistical analyses were performed using the 2‐tailed unpaired Student's *t* test and analysis of variance (ANOVA), with *P* < .05 considered significant.

### 2.11 Cell growth kinetics

To evaluate cell proliferation, cells were counted using a hemocytometer and plated on poly‐L‐ornithine/fibronectin‐coated six‐well plates at 5 × 10^−4^ cells per well. Cell numbers were counted after 0, 2, 3 and 4 days of culture. At each time point, three wells per group were dissociated using Triple‐Select and re‐suspended in growth medium. Aliquots of cells were stained with trypan blue (Sigma‐Aldrich) and counted using a hemocytometer.

### 2.12 BrdU incorporation

Proliferation was assessed using 5‐bromodeoxyuridine (BrdU) incorporation. Cells were plated on glass coverslips coated with poly‐L‐ornithine and laminin and were cultured for 48 hours. The cells were incubated for 4 hours with 10 μmol/L BrdU at 37°C and then fixed with 4% paraformaldehyde for 15 minutes. For immunocytochemical detection of BrdU, the cells were incubated in 2 N HCl for 30 minutes at 37°C and 0.1% Triton X‐100 for 30 minutes, followed by incubation in 0.1 mol/L boric acid (pH 8.0) for 10 minutes. Coverslips were then blocked with 5% normal horse serum in PBS for 1 hour at room temperature, followed by incubation with anti‐BrdU antibody (1:500, BD Biosciences) at 4°C. The cells were then washed with PBS and incubated with a secondary antibody (Alexa Fluor 488 goat anti‐mouse IgG, 1:300, Invitrogen) for 1.5 hours at room temperature. Images were obtained using a Zeiss 880 fluorescence microscope, and BrdU‐labelled cells were counted using a 20× objective.

### 2.13 Statistical analysis

Data are presented as the mean ± SEM. Statistical analysis was performed using one‐factor analysis of variance (ANOVA) followed by a Fisher's LSD (least significant difference) test using the Statistical Analysis System (Enterprise 4.1, SAS Korea). *P* < .05 (*) was considered statistically significant.

## 3 RESULTS

### 3.1 Characterization of YAC128 NSC isolated from the E12.5 embryonic forebrain

In this study, we isolated neural stem cells from the forebrain of E12.5 YAC128 mouse embryos. Neural stem cells were grown as monolayer cultures on poly‐L‐ornithine‐coated dishes (Figure S1A). G‐banding analysis of YAC128 NSCs at passage 7 showed that both WT and TG NSCs had normal karyotypes (Figure S1B). The WT NSCs were female and the TG NSCs were male. The TG mice and cell line were distinguished from the WT mice and cell line by performing PCR genotyping using genomic DNA from mouse tails and the cell lines (Figure S1C). WT and TG PSA‐NCAM^+^ MACS (magnetic‐activated cell sorting)‐sorted cells uniformly expressed NESTIN and SOX2 (Figure S1D), indicating that these cells maintained neural stem cell characteristics over the culture period and that the cell populations were highly homogeneous. Neural stem cells were plated as monolayers and differentiated using a neural differentiation protocol in which the amounts of EGF and FGF‐2 were reduced and the amount of NGF was increased. Differentiated cells expressed neuronal lineage‐specific differentiation markers, including MAP2 and Tuj1 (Figure S1E), demonstrating that the neural stem cells can differentiate into neurons using this protocol. Additionally, using a BrdU incorporation assay, we found that the percentage of proliferating cells in the YAC128 NSCs was significantly higher than in the WT NSCs (Figure S2A,B). YAC128 NSCs also exhibited a higher growth rate compared to the WT NSCs (Figure S2C). The sizes of TG and WT neurospheres were not significantly different (Figure S2D,E), but the radial migration distance of the YAC128 NSCs was significantly increased compared to the WT NSCs (Figure S2F,G).

### 3.2 Expression and cellular localization of mutant Htt aggregates in YAC128 NSCs

**Figure 1:**
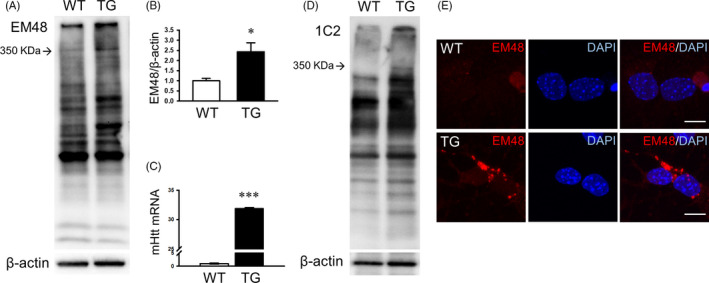
YAC128 NSCs exhibit pathological features of HD. A, B, Representative immunoblot and quantification of EM48 in WT and TG NSCs (DIV7; n = 3). C, Quantitative analysis of mHTT mRNA (n = 3). D, Representative immunoblot of poly glutamine expansion in WT and TG NSCs. E, Immunocytochemistry of EM48 in WT and TG NSCs. Scale bar: 10 µm. Data are shown as the mean ± SEM. *P < .05, ***P < .001 vs WT

The polyglutamine (polyQ) tract in mutant Htt, encoded by expanded CAG repeats, can lead to the formation of toxic oligomers and aggregates.^21^, ^22^ First, we performed Western blot analysis using the mHTT‐specific antibody, EM48. We detected mutant huntingtin proteins of approximately 350 kDa in size and found that they were highly expressed in TG NSCs (Figure 1A,B). Higher levels of mHTT mRNA were detected in the TG NSCs than in the WT NSCs (Figure 1C). Next, we performed Western blot analysis using an antibody against the polyQ tract on exon 1 of mHTT. We found that TG NSCs expressed a polyQ tract of approximately 350 kDa, which was not detected in WT NSCs (Figure 1D). We also performed immunofluorescent staining of the differentiated neurons (DIV7) using the EM48 antibody. We found that EM48‐positive mHTT aggregates were present in the peri‐nucleus of YAC128 NSCs, while aggregates were rarely seen in the WT NSCs (Figure 1E).

### 3.3 Increased intracellular Ca^2+^ and dysfunctional MMP in YAC128 NSCs

**Figure 2:**
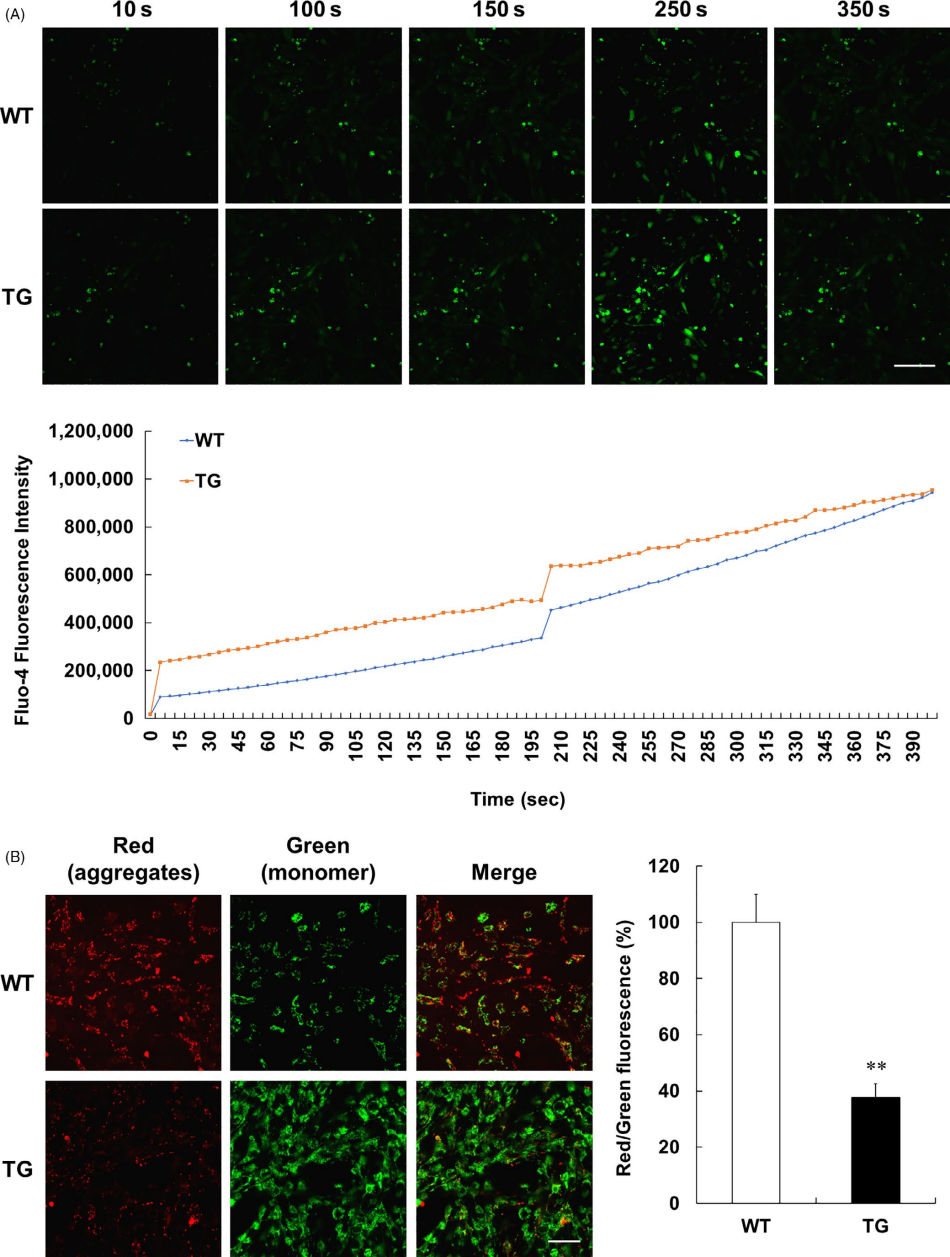
Increased intracellular calcium and MMP dysfunction in YAC128 NSCs. A, Representative images and relative intensity of Ca^2+^ in WT and TG NSCs. Scale bar, 100 μm. Intracellular Ca^2+^ was significantly increased in YAC128 NSCs compared to WT NSCs. B, Mitochondrial membrane potential (MMP, JC‐1 staining) was measured by fluorescence microscopy. Red fluorescence (aggregated form) and green fluorescence (monomeric form) are shown in the representative images. Scale bar, 50 μm. The graph shows the ratio of red/green fluorescence intensity. Values are presented as the mean ± SEM (n = 3 or 5; WT or TG NSCs). **P < .01, compared with WT (ANOVA with Dunnett's test)

To evaluate whether intracellular Ca^2+^ levels are increased in YAC128 NSCs, we performed Ca^2+^ imaging using Fluo‐4 dyes and observed that the intracellular Ca^2+^ level was increased (Figure 2A). To measure MMP in WT and TG NSCs, cells were stained with JC‐1 dye, which exhibits MMP‐dependent accumulation in the mitochondria and changes from green to red fluorescence upon aggregate formation. Therefore, we performed a quantitative analysis of the ratio of red to green fluorescence. In WT NSCs, JC‐1 accumulated as a J‐aggregate (red fluorescence) in the mitochondria, indicating MMP accumulation, whereas TG NSCs exhibited significantly decreased red fluorescence and increased green fluorescence, indicating low MMP. These results indicate a disruption in MMP in the YAC128 NSCs (Figure 2B).

### 3.4 Impaired axonal transport of mitochondria and imbalanced mitochondrial fusion and fission processes in YAC128 NSCs

Recent studies have reported that mitochondrial transport along microtubules is defective in HD primary neurons^23^, ^24^, ^25^, ^26^ and that pathogenic huntingtin inhibits fast axonal transport.^27^ To visualize mitochondrial movement, live cell imaging was performed using MitoTracker (Ds‐Red) in the NSCs (DIV7). We found that YAC128 NSCs exhibited reduced mitochondrial movement compared to WT NSCs.

**Figure 3:**
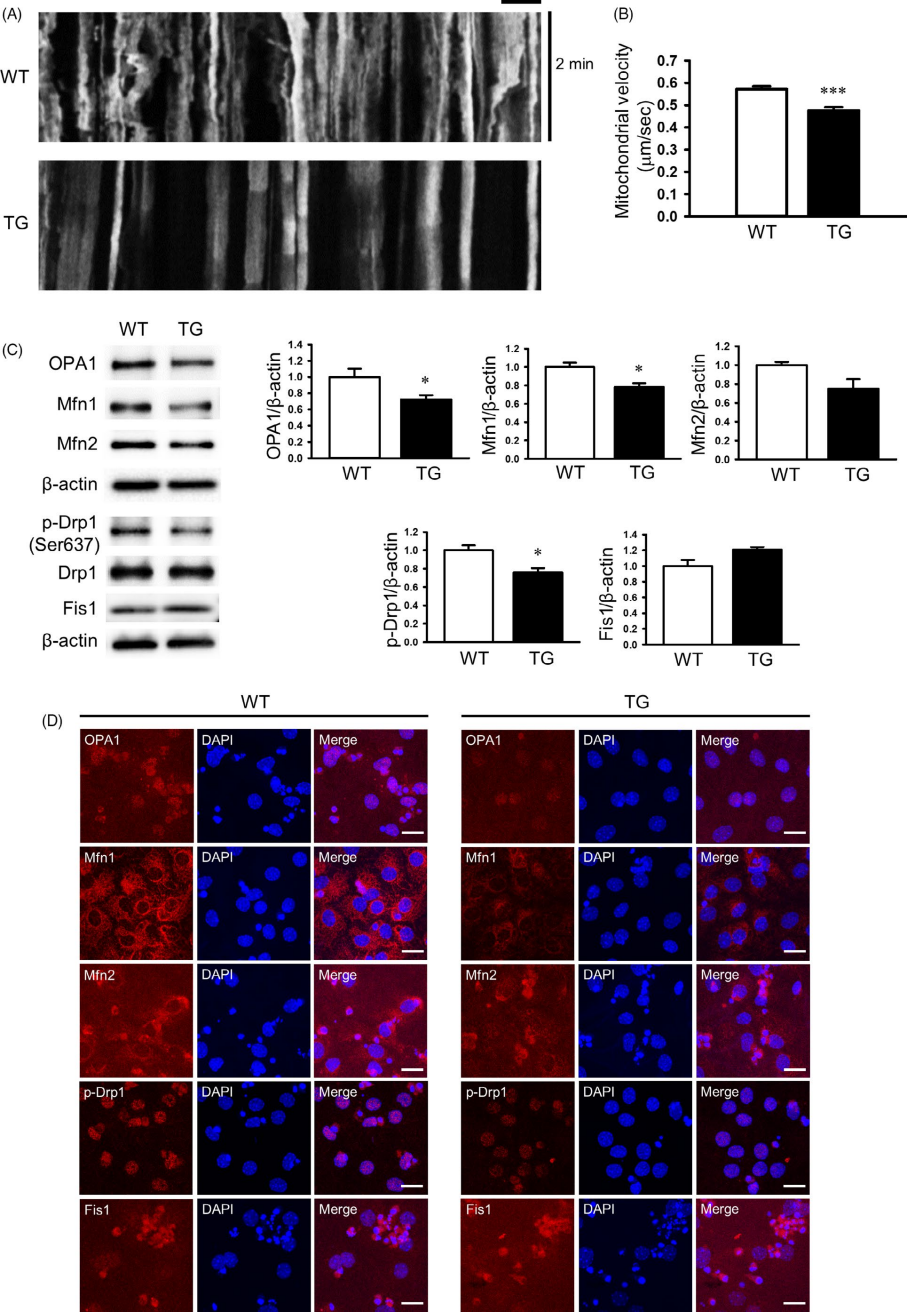
Impaired mitochondrial dynamics and impaired balance of mitochondrial fusion and fission in YAC128 NSCs. A, Representative kymographs of mitochondrial movements. Scale bar, 10 µm. B, Average velocity was analysed in the motile mitochondria from differentiated NSCs. C, Western blot analysis of mitochondrial dynamics‐related proteins: fusion proteins (OPA1, Mfn1, Mfn2); fission proteins (Drp1, Fis1). Quantification and comparison of protein expression levels (n = 3). D, Immunofluorescence analysis of the expression of mitochondrial dynamics‐related proteins in WT and TG NSCs. Scale bar, 20 µm. Data are presented as the mean ± SEM.*P < .05, **P < .01, ***P < .001 to WT.

Time‐lapse image recordings were made to generate kymographs, and quantitative analysis of mitochondria velocity was performed using IMARIS. Velocity was significantly decreased in the YAC128 NSCs (DIV7) (Figure 3B). Several studies using HD cell lines and primary neurons that express mHTT have suggested that mitochondrial dynamics are imbalanced in HD.^23^, ^25^, ^28^, ^29^ In mammals, OPA1, mitofusin 1 (Mfn 1) and mitofusin 2 (Mfn 2) regulate fusion, whereas dynamin‐related protein 1 (Drp1) controls mitochondrial fission.^30^, ^31^, ^32^ Drp1 is a member of the dynamin superfamily of GTPases, and it has three phosphorylation sites. When phosphorylated at Ser616 and Ser585, Drp1 stimulates mitochondrial fission. In contrast, when Drp1 is phosphorylated at Ser637, mitochondrial fission is inhibited. Western blot analysis revealed that the expression of OPA1, Mfn1 and pDRP1 (Ser 637) was significantly decreased in YAC128 NSCs. Expression of Mfn2 was decreased, and the expression of Fis1 was increased, but these changes were not statistically significant (Figure 3C). Immunocytochemical analysis further confirmed that the expression of p‐Drp1 (Ser 637), OPA1 and Mfn1 was significantly decreased in the YAC128 NSCs (Figure 3D). These results suggest that the pathogenic mHTT proteins may disrupt mitochondrial transport via imbalanced mitochondrial dynamics (*ie* increased fission and decreased fusion) in YAC128 NSCs.

### 3.5 Decreased voltage‐gated Na^+^ currents in YAC128 NSC

**Figure 4:**
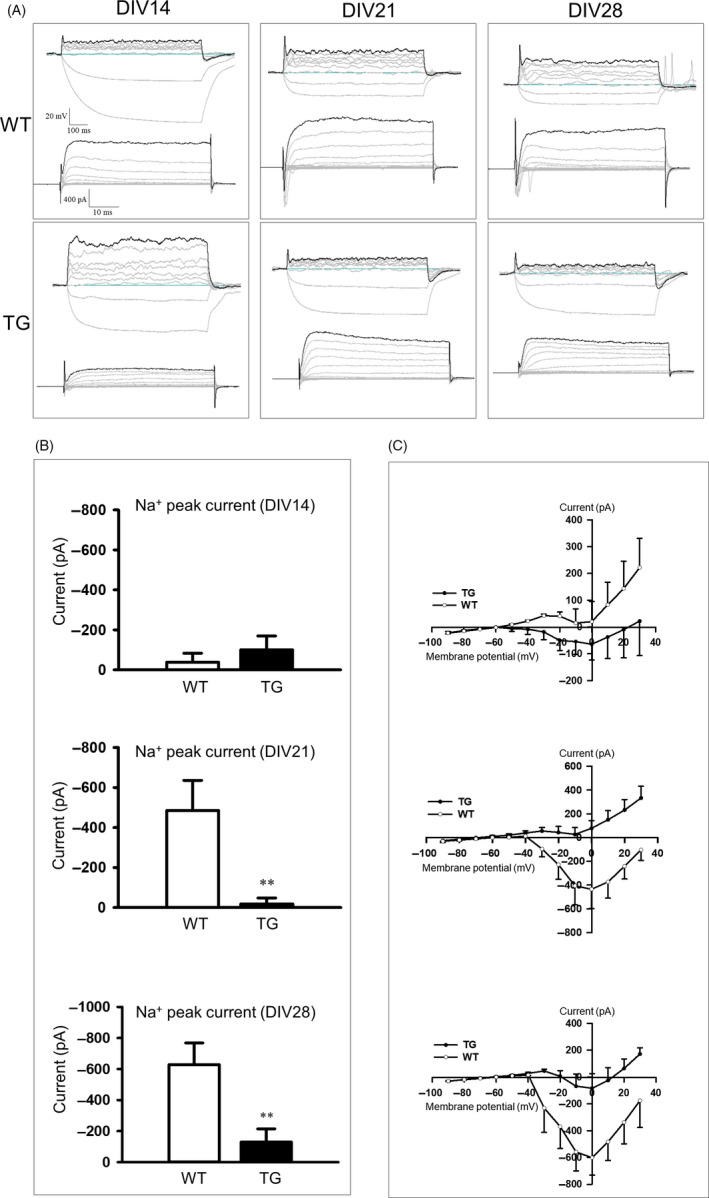
Electrophysiological properties of YAC128 NSCs. A, Traces of current‐clamp and voltage‐clamp modes in WT and TG NSCs at the differentiation stages of DIV14, DIV21 and DIV28 are shown. A regenerative action potential (single) in the current‐clamp mode at DIV14 and DIV21 (upper traces) was produced in WT and TG NSCs. Amplitude of the inward voltage‐gated sodium current in the voltage‐clamp mode at DIV14, DIV21 and DIV28 (lower trace, holding potential = 60mV) was produced in the WT and TG NSCs. B, Sodium peak current was significantly decreased in the TG NSCs at DIV21 and DIV28 (n = 6, **P < .01 to WT). C, Sodium currents are shown at DIV14, DIV21 and DIV28 for WT and TG NSCs (n = 6). The sodium current was significantly decreased in the TG NSCs at DIV21 and DIV28. Data are shown as the mean ± SEM

In order to investigate the extent of neuronal maturation, we performed whole‐cell patch‐clamp analysis on the YAC128 NSCs. Cells that lack regenerative spikes (Passive AP) and those with weak regenerative responses (Abortive AP) are less mature than cells with full‐size AP. Also, cells that lack the ability to fire repetitively (Single AP) are less mature than cells which have developed that ability (Repetitive AP).^19^, ^20^ YAC128 WT and TG NSCs showed similar maturation profiles (Abortive AP). We monitored the extent of the action potentials at DIV14, DIV21 and DIV28. We focused on the capability of cells to elicit the appearance of voltage‐gated Na^+^ currents. In Figure 4A, the upper tracing shows that both cell types generated similar action potentials, and the lower tracing shows the voltage‐gated currents elicited at the step current from a holding voltage of −60 mV. In each case, samples were obtained from YAC128 WT and TG NSCs at DIV14, DIV21 and DIV28. Both WT and TG NSCs showed high voltage‐gated Na^+^ currents at DIV21 and DIV28. It was evident that the inward Na^+^ current varied substantially from cell to cell. We found that the peak Na^+^ current was significantly decreased in YAC128 NSCs compared to WT NSCs at DIV 21 (*P* = .036) and DIV 28 (*P* = .017; Figure 4B). The sodium current was also decreased in the YAC128 NSCs at DIV21 and DIV28 (Figure 4C) compared to WT NSCs. These results suggest that the extent of neuronal maturation in WT NSCs is greater than in TG NSCs. No significant differences in K^+^ current between the WT and YAC128 NSCs were detected (data not shown).

### 3.6 Defective ubiquitin‐proteasome and autophagy systems in YAC128 NSCs

**Figure 5:**
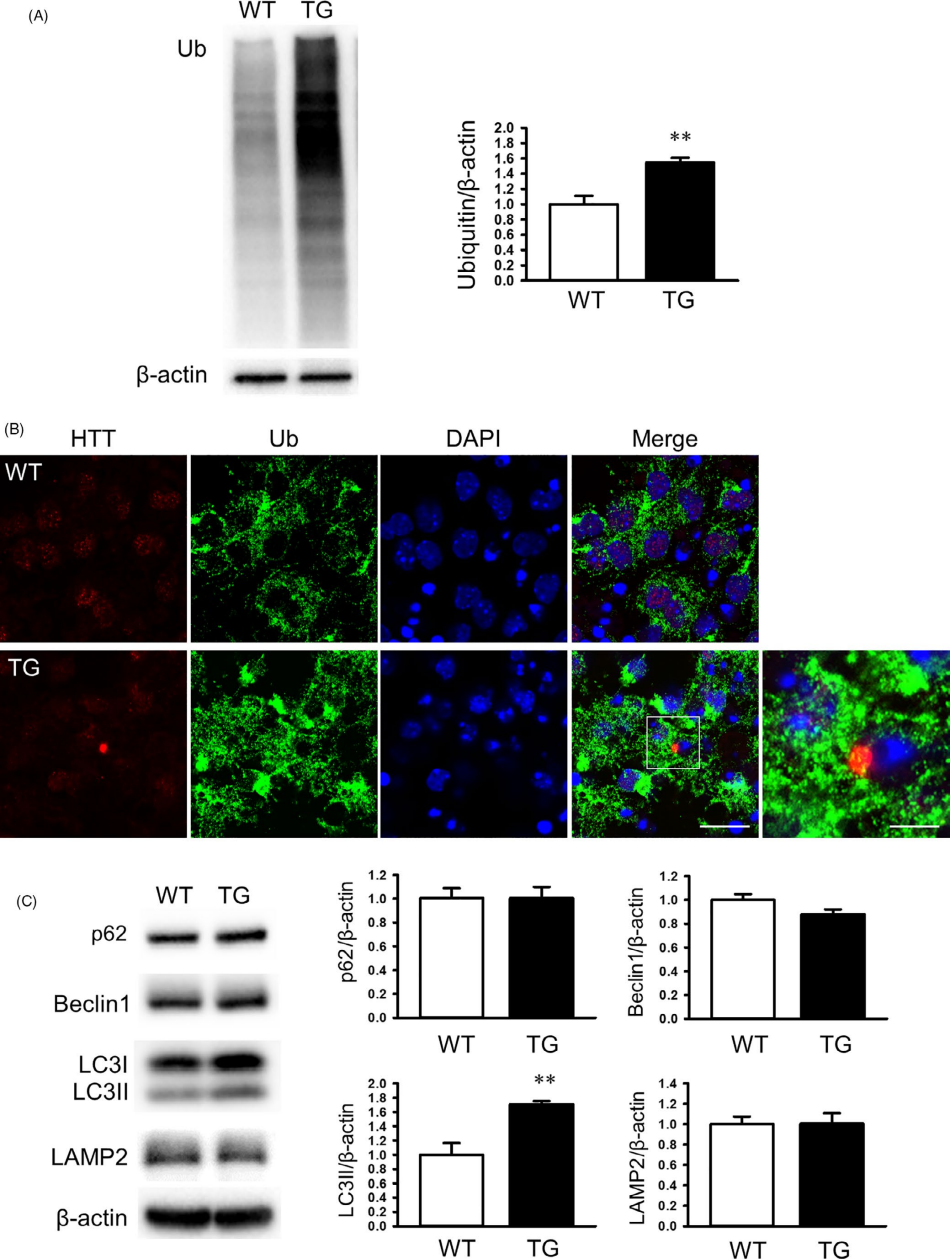
Impairment of the ubiquitin‐proteasome and autophagy systems in YAC128 NSC. A, Representative immunoblot showing the expression of ubiquitin in WT and TG NSCs (DIV7, n = 3). B, Immunofluorescence analysis of the expression of HTT (red fluorescence) and Ub (green fluorescence) in WT and TG NSCs (DIV7). Scale bar, 20 or 10 µm. C, Representative immunoblot showing the expression of autophagy‐related proteins in WT and TG NSCs (DIV7, n = 3). Data are shown as the mean ± SEM. **P < .01 compared with WT NSC

Dysfunction in the ubiquitin‐proteasome system is known to occur in HD.^33^, ^34^ Additionally, defects in autophagy are also known to occur in HD,^35^ which lead to a build‐up of toxic materials in the cytoplasm and empty autophagosomes. To examine the functions of the autophagy and ubiquitin protease systems in our differentiated NSCs, we measured the expression of Ub (ubiquitin), p62 (SQSTM1, sequestosome 1; cargo protein marker), Beclin1 (autophagosome membrane formation related protein), LC3b (microtubule‐associated protein light chain 3, autophagosome formation marker) and LAMP2 (lysosome‐associated membrane protein). Western blot analysis showed that the expression of Ub was significantly increased in the TG cells compared to the WT cells (Figure 5A), suggesting that UPS‐mediated degradation is impaired in the TG NSCs (DIV7). To examine whether Ub expression is increased and, at the same time, whether HTT aggregates are co‐expressed with Ub in YAC128 NSCs, we performed double immunostaining using HTT‐ and Ub‐specific antibodies. mHTT aggregates and Ub aggregates were detected in YAC128 NSCs (DIV7; Figure 5B). Interestingly, we found that mHTT aggregates were co‐expressed with Ub aggregates, suggesting that impairment of UPS‐mediated degradation may inhibit the clearance of mHTT aggregates. The expression of LC3II, but not p62, was significantly increased in YAC128 NSCs compared to WT NSCs (Figure 5C). LAMP2 expression was not significantly different between YAC128 NSCs and WT NSCs (Figure 5C). Taken together, these results suggest that the ubiquitin‐proteasome system and autophagy‐related clearance are impaired in YAC128 NSCs.

### 3.7 Elevated phospho‐tau level in YAC128 NSCs

Tauopathy has been reported to be involved in Huntington's disease,^36^ and Tau hyperphosphorylation and aggregation is strongly associated with neuronal dysfunction and progressive neuronal death in HD. To determine if mutant huntingtin aggregates correlated with tauopathy in the YAC128 NSC model, we measured the expression of phospho‐PHF‐tau (Ser202/Thr205) and total Tau (Tau5). We found that the ratios of AT8 and Tau5 were significantly increased in the YAC128 NSCs (Figure S3).

## 4 DISCUSSION

In an attempt to develop a reliable cellular model of HD, we have established a new neural stem cell line from the forebrain of E12.5 embryos from YAC128 transgenic mice, one of the most commonly used mouse models of HD. First, we demonstrated that mutant huntingtin (mHTT) protein was highly expressed in YAC128 NSCs and that EM48‐positive mHTT aggregates were found in the perinuclear region. Proteins which interact with mutant huntingtin aggregates contain ubiquitin‐associated motifs. When they are recruited to the aggregates, cellular dysfunction results.^37^ Misfolded huntingtin aggregates may impair autophagic function and therefore promote the accumulation and toxicity of aggregates, while autophagy induction enhances the clearance of mutant Htt protein and plays a protective role.^38^

We also found that Ca^2+^ levels and ROS signals were significantly increased in YAC128 NSCs. Previous studies have shown that region‐specific oxidative damage in the brain is present in HD patients,^39^ which contributes to neuronal loss.^40^ It has been reported that excessive ROS and mitochondrial calcium uptake lead to elevated oxidative stress in YAC128 HD embryonic fibroblasts.^16^ Since calcium and ROS signals are enhanced in YAC128 mice, adult neural progenitor cells from the YAC128 mouse brain show increased proliferation and migration.^15^ Here we demonstrated that YAC128 NSCs have higher levels of Ca^2+^ and ROS compared to WT NSCs.

Additionally, mitochondrial dynamics were defective in the YAC128 NSCs. Mitochondrial dysfunction is a key event in HD, and this includes defective mitochondrial axonal transport and abnormal fusion and fission.^18^, ^23^, ^41^, ^42^ mHTT interacts with the mitochondrial protein dynamin‐related protein 1 (Drp1), enhances Drp1 GTPase enzymatic activity and causes excessive mitochondrial fragmentation and abnormal distribution, leading to defective mitochondrial axonal transport and selective synaptic degeneration. Our results demonstrate that mitochondrial axonal transport velocity is significantly decreased in the YAC128 NSCs compared to the WT NSCs. Mitochondrial fusion‐ and fission‐associated genes were also altered, and an increase in mitochondrial fission and decrease in fusion were observed in the YAC128 NSCs.

Electrophysiological analysis demonstrated that the capacity of the NSCs to differentiate into functional neurons was significantly reduced in the YAC128 NSCs. In this study, we employed the whole‐cell patch‐clamp technique to monitor the voltage response variability and Na^+^ current amplitude at various stages of neuronal differentiation. Mouse embryonic stem cells have been previously shown to differentiate into functional GABAergic neurons efficiently under defined in vitro conditions, and these neurons can survive and differentiate after delivery to a mouse model of HD.^43^ Altered excitatory and inhibitory inputs to pyramidal neurons in the cortex appear to be prevailing deficits in HD. Our results showed that the peak Na^+^ current was significantly decreased in YAC128 NSCs compared to WT NSCs. It was evident that the extent of neuronal maturation in WT NSCs is greater than in TG NSCs.

We also observed that the ubiquitin‐proteasome and autophagy systems were impaired in YAC128 NSCs. The expression of ubiquitin was significantly increased in YAC128 NSCs, leading to impairment of UPS‐mediated degradation. This allows mHTT proteins to accumulate into insoluble, ubiquitinated aggregates. Failure of cargo is responsible for inefficient autophagy in HD.^44^ We also measured the expression of LC3, an autophagosome marker, and found that LC3II levels were significantly increased in YAC128 NSCs. However, LAMP2 expression did not change in the TG NSCs, suggesting that autophagy was impaired. These results suggest that autophagic dysfunction in YAC128 NSCs can lead to the accumulation of mHTT aggregates.

Lastly, we found that AT8 and Tau5 were significantly increased in YAC128 NSCs. It has been previously reported that HD pathology may promote tau hyperphosphorylation and induce tau pathology. Tau is a microtubule‐associated protein widely expressed in the central nervous system, and it regulates microtubule dynamics, neurite outgrowth and axonal transport^45^ in neurons. Tau hyperphosphorylation and aggregation is strongly associated with neuronal dysfunction and progressive neuronal death.^46^ Tau hyperacetylation‐impaired microtubule assembly can promote tau fibrillization in vitro.^47^ It has been reported that acetylation and phosphorylation of tau at multiple sites may act synergistically in the pathogenesis of tau fibrillization. Tau phosphorylation (AT8) was enhanced in YAC128 NSCs compared to WT NSCs. Therefore, it is likely that tauopathy contributes to HD pathology.^36^

Recently, the use of several induced pluripotent stem cell (iPSC) models^33^, ^34^, ^44^, ^48^, ^49^, ^50^, ^51^, ^52^, ^53^, ^54^, ^55^, ^56^, ^57^, ^58^ or directly differentiated neurons^55^ from HD patients have been reported, which will more accurately represent the pathophysiology of HD patients than murine HD NSC models.^8^, ^15^ However, differentiation of HD patient‐derived iPSCs into mature neurons is time‐consuming, taking at least 10 weeks. Furthermore, it is very difficult to observe the expression of pathological markers in these cells even after differentiation, such as the formation of mHTT aggregates, as this is also dependent upon the number of CAG repeats present. In contrast, our results demonstrate that neural stem cells derived from YAC128 mice rapidly express HD pathological markers, even at the undifferentiated stage. This provides strong advantages over human iPSC models for practical purposes, such as drug screening.

In summary, we have established a new neural stem cell line from the forebrain of YAC128 transgenic mice (E12.5), one of the most commonly used mouse models of HD. Our findings suggest that YAC128 NSCs exhibit various pathological features of HD. Therefore, this new cell line will be useful for studying HD pathogenesis and for future drug screening.

## CONFLICT OF INTEREST

JS is the founder and CEO of iPS Bio, Inc. The other authors declared no conflicts of interest.

## AUTHOR CONTRIBUTIONS

EL HRP, CPH and JS were responsible for the study concept and design. EL, CPH, YK, JC, SL, HJP and BL were responsible for data acquisition. EL, HRP, CPH, YK, TAK, SJK, HSK and JS performed data analysis and manuscript writing. JS provided financial support and finalized the manuscript. EL HRP and CPH contributed equally.

## Supporting information

Figures S1‐S3

## Data Availability

The data that support the findings of this study are available from the corresponding author upon reasonable request.
