# Supplementary material for: Neural stem cells derived from the developing forebrain of YAC128 mice exhibit pathological features of Huntington’s disease
Source: Cell Prolif. 2020 Aug 31;53(10):e12893. doi: 10.1111/cpr.12893 (PMC7574873; doi:10.1111/cpr.12893)
Supplement: Supplementary file 1 — Figures S1‐S3 [file CPR-53-e12893-s001.docx]

**Supporting information file**

**Cell Proliferation**

**Neural stem cells derived from the developing forebrain of YAC128 mice exhibit pathological features of Huntington’s disease**

Endan Li^1,§^, Hee Ra Park^2,§^, Chang Pyo Hong^3,§^, Younghoon Kim^1^, Jiwoo Choi^1^, Suji Lee^1^, Hyun Jung Park^1^, Bomi Lee^2^, Tae Aug Kim^1^, Seong Jin Kim^3^, Hyun Sook Kim^4^ and Jihwan Song^1,2,*^

**
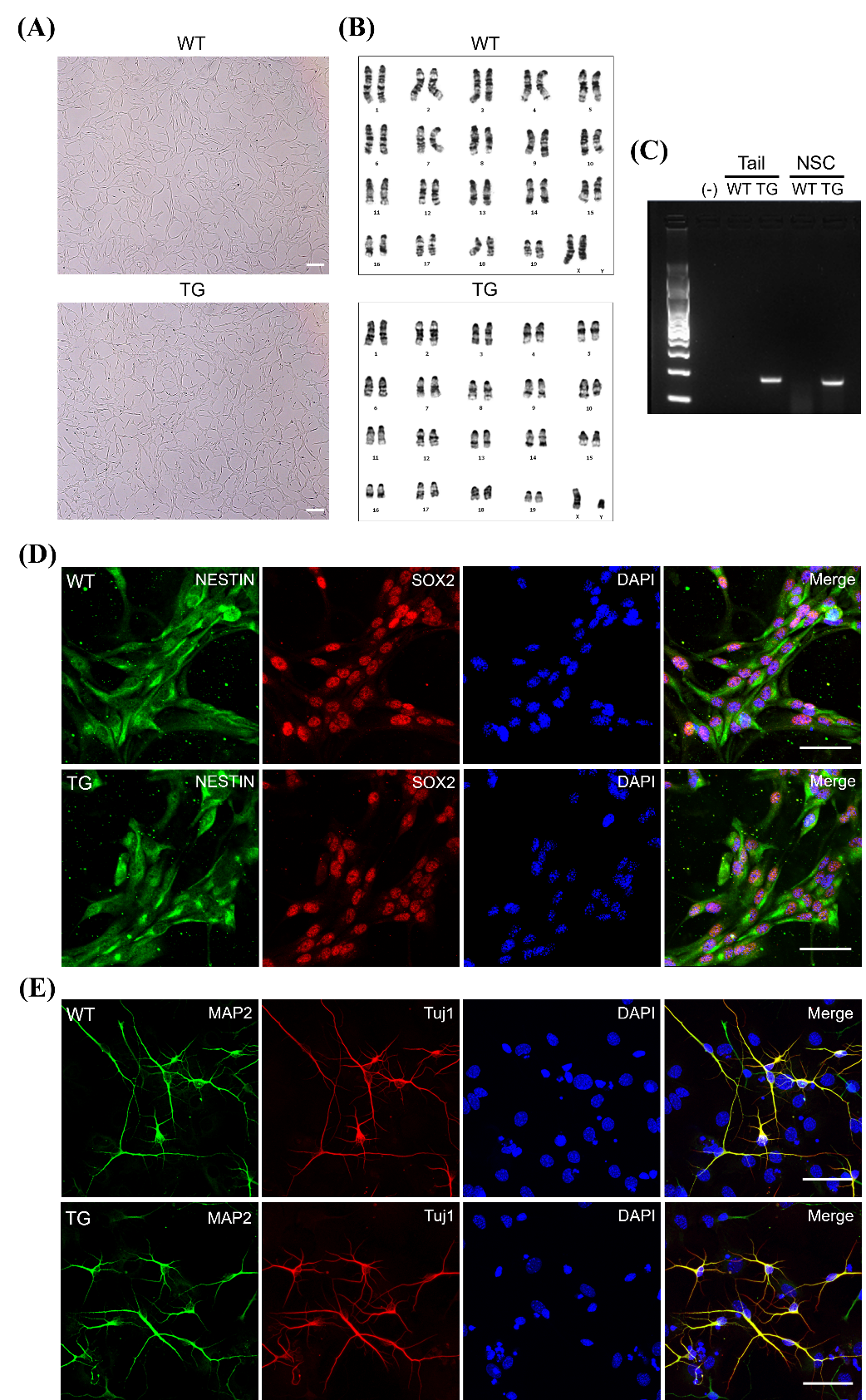
**

**Figure S1. Characterization of neural stem cells derived from the forebrain of YAC128 embryo (E12.5). (A)** WT and TG NSCs were grown as monolayers in poly-ornithine/fibronectin double-coated dish. Scale bar, 100 µm. **(B)** Karyotyping results from WT and TG NSCs. **(C)** Genotyping results from WT and TG NSCs. Nuclease-free water in lane 1 was used as a negative control. Genomic DNAs of tails from WT and TG mice shown in lane 2 and lane 3 were used as a positive control. PCR genotyping from genomic DNAs of NSCs from WT and TG in lane 4 and lane 5 are presented. These results indicate that WT and TG genotypes can be distinguished by genotyping using both tail and NSC samples. **(D)** NESTIN (green) and SOX2 (red) immunoreactive cells can be found in YAC128 NSC grown in poly-ornithine / fibronectin-coated dishes. Scale bar, 20 µm. **(E)** Differentiated YAC128 NSC (DIV7) expressed neuronal markers, such as MAP2 (green) and Tuj1 (red). Scale bar, 50 µm.

**
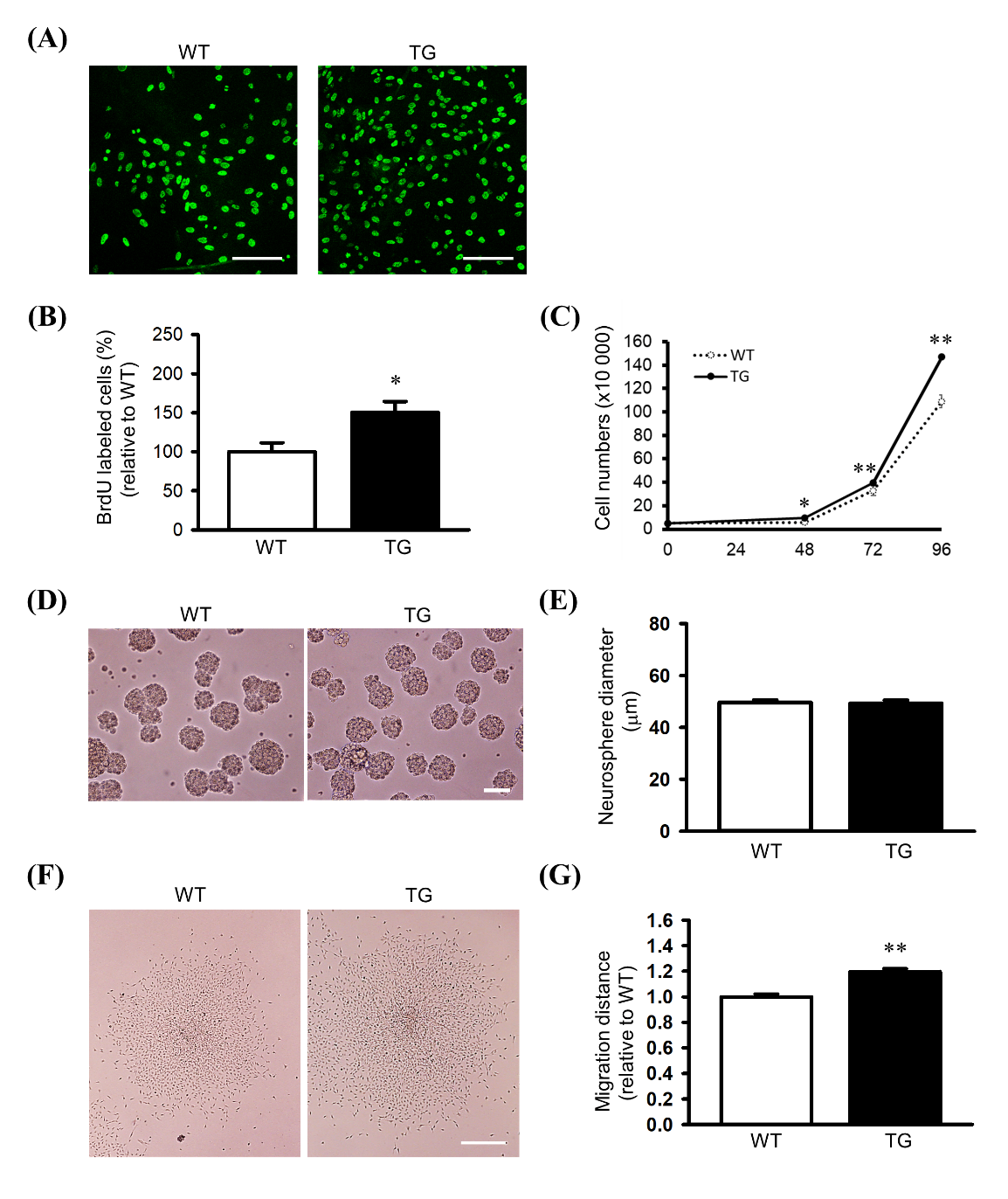
**

**Figure S2. YAC128 NSC derived from the forebrain of embryo (E12.5) exhibit increased proliferation, migration compared with WT NSC. (A, B)** BrdU incorporation assay. The percentage of BrdU-positive cells in YAC128 NSC was significantly increased relative to WT NSC (*n*=4, **P* < 0.05). Scale bar, 100 µm. **(C)** YAC128 NSC exhibited a higher growth rate compared with WT NSC (*n*=3, **P* < 0.05, ***P* < 0.01). **(D, E)** Neurosphere assay. The size of neurospheres was measured after 2-day culture. Scale bar, 100 µm. **(F)** Neurospheres cultured on poly-L-ornithine- and laminin-coated dishes migrated outwards radially. Scale bar, 25 µm. **(G)** The radial migration distance of neurospheres in YAC128 NSC was significantly increased, compared with WT NSC after 12-hour cultivation (n=50, ***P* < 0.01).

**
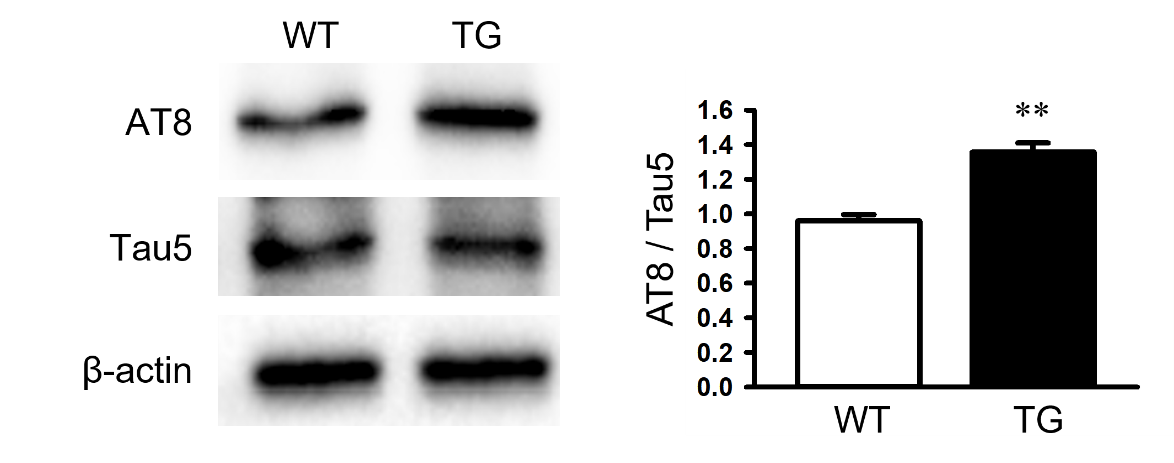
**

**Figure S3. Increased phospho-tau level in YAC128 TG NSC.**

Representative immunoblot showing the expression of phospho-PHF-tau (AT8) and total Tau (Tau5) in WT and TG NSCs. Quantification and comparison of protein expression levels (*n*=3). Data are shown as the mean ± SEM. ***P*< 0.01 compared with WT NSC.
